# Management of Heel Pad Degloving Injury After Severe Foot Crush Injury: A Case Report Study

**DOI:** 10.7759/cureus.14191

**Published:** 2021-03-30

**Authors:** Dimitrios Giotis, Chris Kotsias, Sotiris Plakoutsis, Michael-Alexander Malahias, Christos Konstantinidis

**Affiliations:** 1 Orthopaedic Department, General Hospital of Ioannina "G. Chatzikosta", Ioannina, GRC; 2 Department of Orthopaedics and Traumatology, Clinica Ars Medica, Gravesano, CHE

**Keywords:** degloving injury, crush injury, heel pad, negative pressure wound therapy, skin graft

## Abstract

Crush injuries of the foot and ankle are uncommon and they have a poor prognosis leading to some form of disability. Degloving injuries of the heel and foot after crush injuries are rare and very challenging to manage due to the need for reconstruction of both osseous and soft tissue architecture. We present a salvage strategy for an open injury to the foot with extensive soft tissue detachment from the plantar and dorsal surface. A 30-year-old man was transferred to the Emergency Department from a neighboring hospital with a crush injury to the foot that had resulted in a degloving injury of the heel pad, after a motorcycle accident. The patient had a 20 cm circumferential wound that was extending from dorsal to the plantar surface along with rupture of the extensor digitorum longus (EDL) tendons and transection of the superficial peroneal nerve. There was an extensive detachment of soft tissues from the deep fascia and bones, whereas the posterior tibial artery was intact. In radiographic imaging, a small inferior avulsion fracture of the calcaneus along with fractures of the cuneiform bones was revealed. The initial management involved thorough surgical debridement, removal of necrotic tissues, repair of EDL tendons and fracture stabilization. Negative-pressure wound therapy was also applied for six weeks. Subsequently, a split-thickness skin graft was used to cover the skin defect. Six months after injury, the patient had a normal range of motion, intact sensation over the sole and could ambulate independently. Although the majority of heel pad degloving injuries have a poor prognosis, there are positive prognostic factors as presented in the current case for a satisfying functional final outcome, which include vascular intergrity, fracture stabilization, soft-tissue reconstruction with negative pressure wound therapy, and application of skin grafts.

## Introduction

Degloving of the foot involving the heel pad is an uncommon injury mainly resulting from high-energy lower limb trauma such as crush injuries and road traffic accidents [1]. It is characterized by detachment of subcutaneous soft tissue from surrounding elements, which can be accompanied by tendon and nerve transection, osseous defects and vascular damage compromising the viability of skin and soft tissues, thus leading to bacterial infection and liquefactive necrosis [2]. This kind of injury is frequently related to severe morbidity and poor prognosis resulting in some form of disability that could have an impact on the quality of a patient's life [3].

Therefore, the management of these foot injuries is challenging and might involve the restoration of both bony and soft tissue architecture, without any delay [3]. The anatomy of the area along with certain difficulties in foot skin coverage and postoperative weight-bearing are factors that should be taken into consideration [3-5]. Although there are no standardized protocols, in cases in which there is adequate blood supply, debridement and reattachment of the avulsed flap might be sufficient [4-6]. In contrast, in more complicated cases, where extensive separation is present, split- and full-thickness grafts and flaps along with microvascular anastomoses might be needed [3-7].

The aim of the present study was to report the salvage strategy for repair of an open-degloving injury to the foot, including heel pad with extensive soft tissue detachment from both plantar and dorsal sides. The role of soft tissue reconstruction with the use of negative-pressure-wound therapy and subsequent split-thickness graft is also emphasized.

## Case presentation

A 30-year-old male with a body mass index (BMI) 24.7 kg/m^2^, was transferred to the Emergency Room (ER) from a neighboring hospital after a motorcycle accident. He had sustained a crush injury to his left foot with an open-degloving of the heel pad. In the emergency room, the patient was conscious (Glasgow Coma Scale = 15) and hemodynamically stable and no further injuries were observed. Apart from his statement that he was a tobacco smoker, no previous medical conditions were reported.

**Figure 1:**
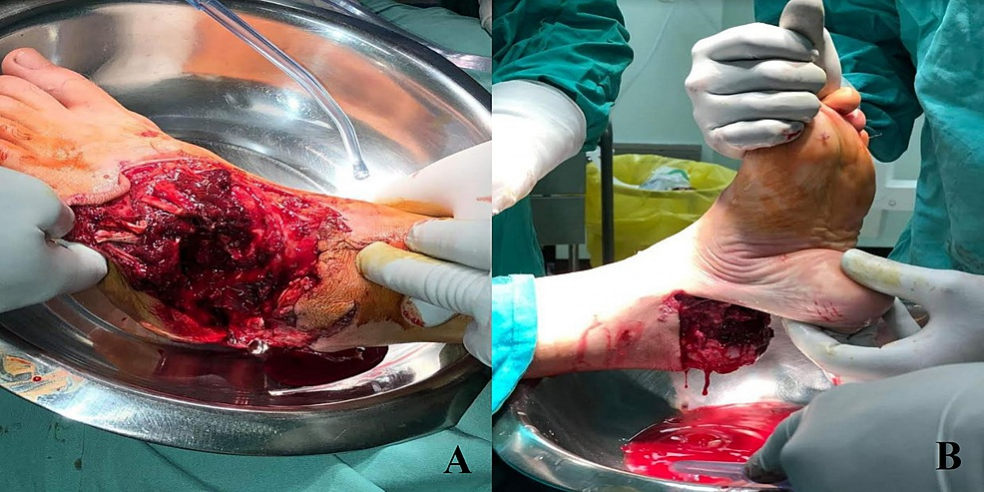
Degloving injury of the left foot including the heel pad.

Clinically, a 20 cm circumferential wound was found, which was extended from the dorsal surface of the mid-foot to the plantar surface of the hind-foot, including the heel pad. Extensive detachment of soft tissues from the deep fascia and bones was visible. The patient could not actively extend his toes and there was an almost complete lack of sensation over the dorsal surface. The posterior tibial artery was palpable. However no sign of dorsalis pedis pulse could be detected (Figures 1A, 1B).

**Figure 2:**
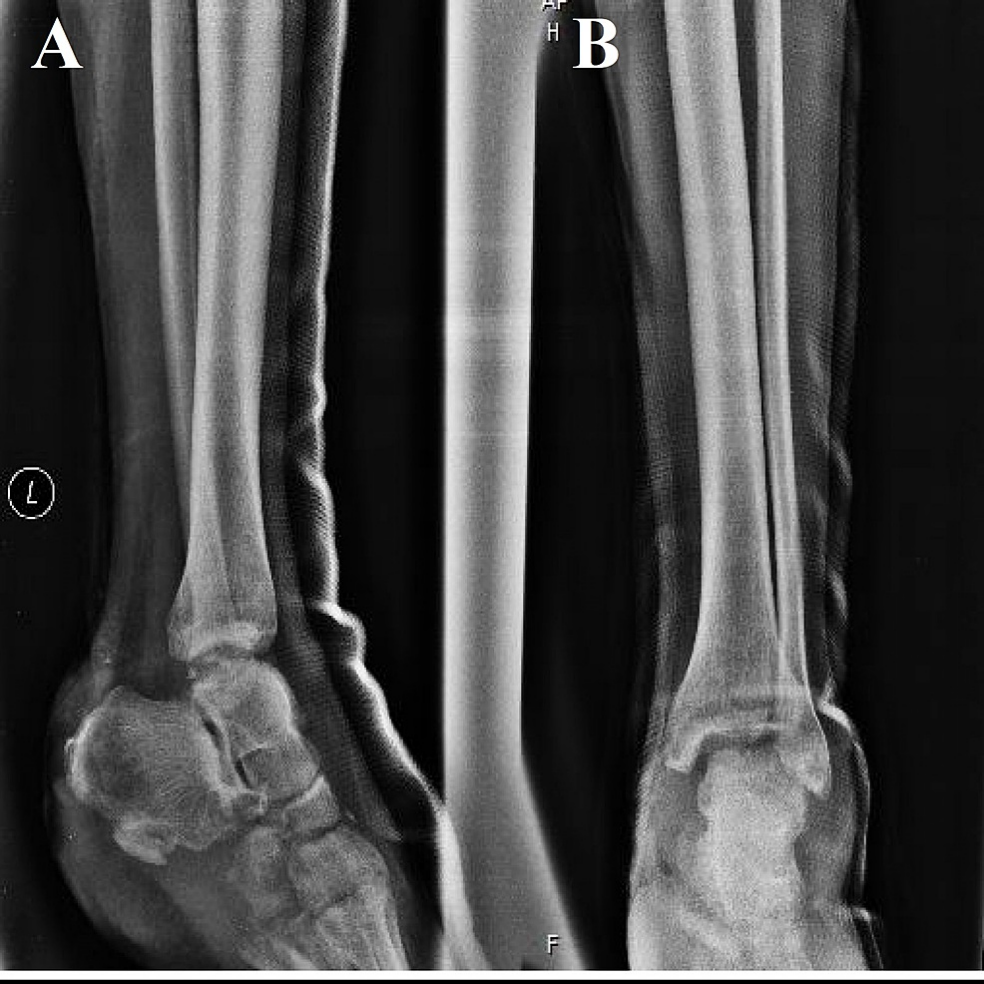
Anteroposterior (A) and lateral (B) radiograph displaying the avulsion fracture of the calcaneus and the fractures in the cuboid and cuneiform bones.

**Figure 3:**
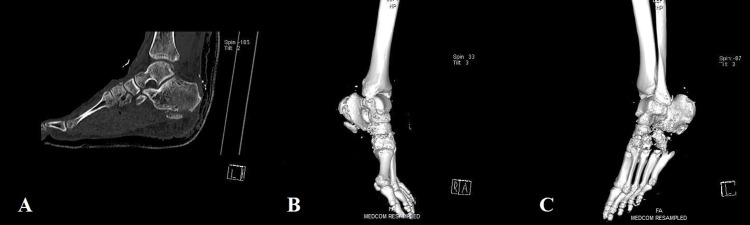
CT of the foot and ankle (sagittal view [A] and reconstructed images [B, C]) showing the osseous lesions.

Radiographically, a small avulsion fracture from the inferior part of the calcaneus along with fractures in the cuboid and cuneiform bones were found (Figures 2A, 2B). In order to possess an improved view of the skeletal injury, a computed tomography (CT) scan was also executed (Figures 3A-3C). Overall, based on the clinical and radiological findings, the present foot open fracture was classified as a Gustilo-Anderson IIIB injury.

Primary management in the ER started with wound cleansing and dressing with moist saline gauze. Cultures were obtained from the open wound and a short posterior splint was placed. Preoperatively, broad-spectrum antibiotics, according to the antibiotic protocol for open fractures in our department, comprised cefoxitin and amikacin along with tetanus immunoglobulin were administered. In the operating room, under general anesthesia, meticulous inspection of the wound and diligent evaluation of the surrounding structures were performed. It was observed that there was an extensive circumferential detachment of soft tissues especially in the heel pad where both skin and plantar fat were separated from the calcaneus. Furthermore, extensor digitorum longus (EDL) and peroneus brevis (PB) tendons were ruptured and the dorsal branch of the superficial peroneal nerve was also cut. In addition, the presence of avulsion fracture in the calcaneus and the fractures of cuboid and cuneiform bones were also confirmed intraoperatively but the ankle joint stability that was also assessed was found intact.

**Figure 4:**
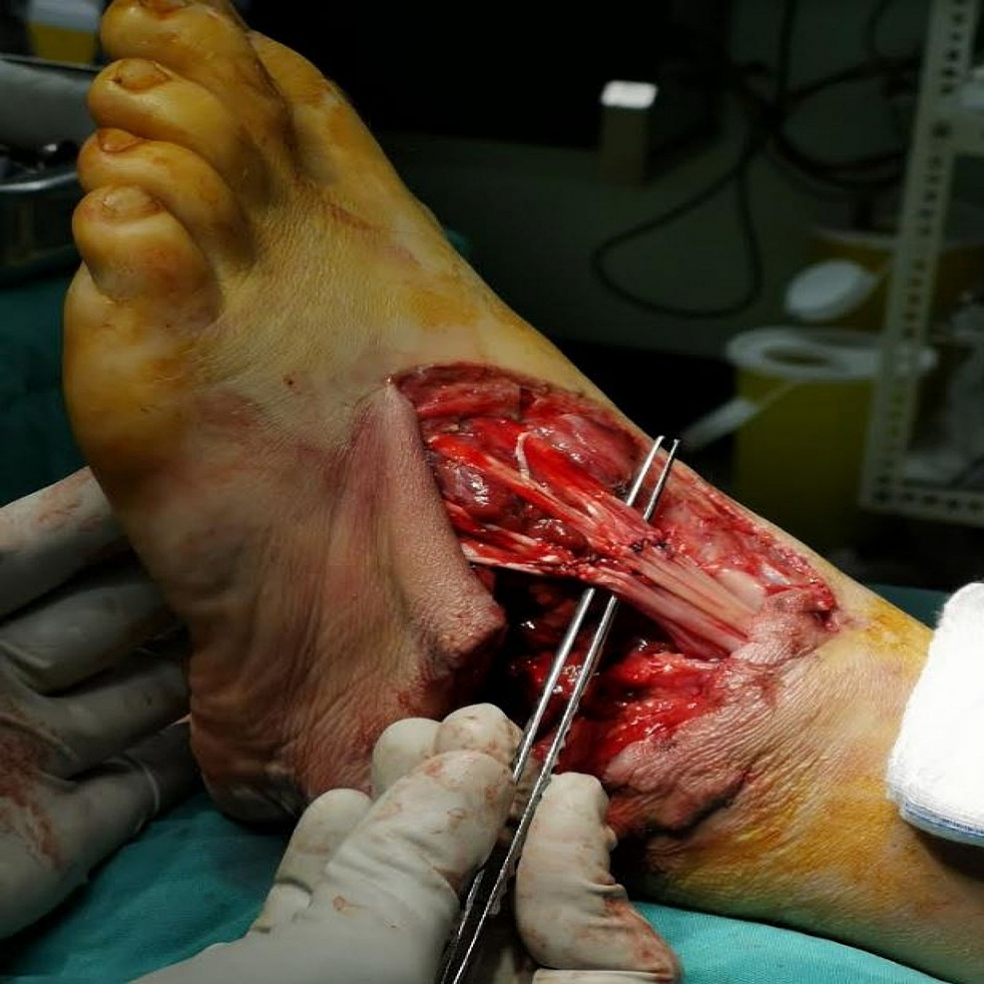
Intraoperative view demonstrating tendons` repair.

Prior to any therapeutic intervention, intraoperative cultures were also obtained. Initially, the wound was radically debrided and lavaged with nine litres of normal saline 0.9%. Any nonviable and necrotic soft tissues, including bone fragments and part of the dorsal skin, were excised. After the thorough debridement and irrigation, the EDL tendons were repaired whilst the proximal part of the PB tendon was sutured on the peroneus longus (PL) tendon. The fractures were stabilized by soft tissue and viable skin reapproximation with nylon sutures under gentle tension. An effort to cover the repaired tendon with remaining viable skin was performed. Finally, a negative-pressure-wound-therapy device was applied along with a splint in 90 degrees position (Figure 4).

**Figure 5:**
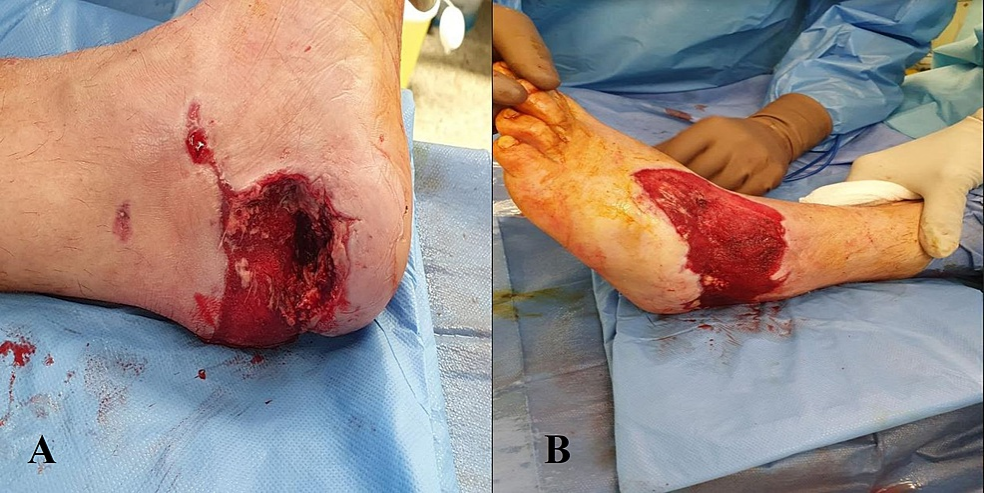
Five weeks post-operatively after VAC application. VAC - vacuum-assisted closure

Enterococcus faecalis contamination was indicated in the antibiogram and therefore the antibiotic therapy was changed to vancomycin according to the individual susceptibility report. Periodic debridement with removal of necrotic tissues and irrigation were executed twice per week when the negative pressure dressing was reapplied. Six weeks post-operatively, vacuum-assisted closure (VAC) device and splint were removed. Passive motion of ankle joint was allowed as tolerated. Two weeks later, the new wound cultures that were acquired were negative and thus a split-thickness skin graft was placed to cover the existing skin defect (Figures 5A, 5B).

**Figure 6:**
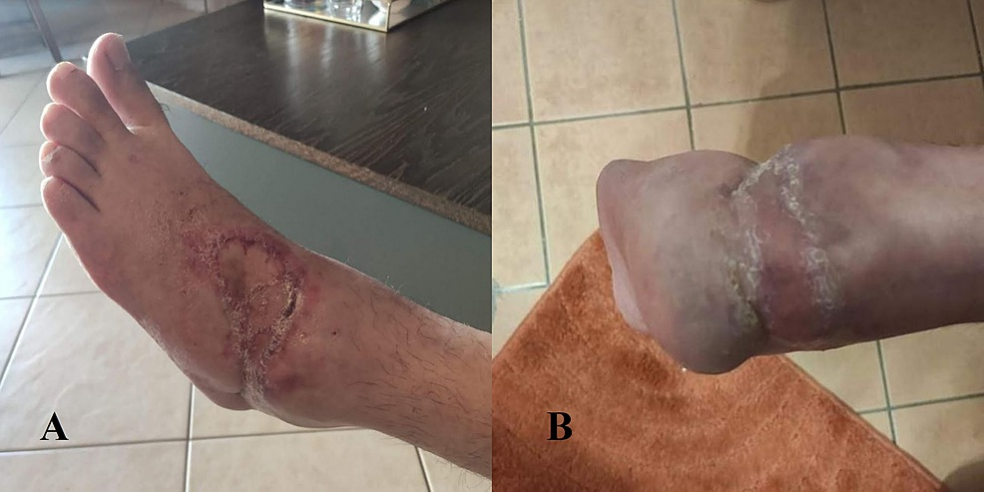
Final cosmetic and functional result six months post-operatively.

**Figure 7:**
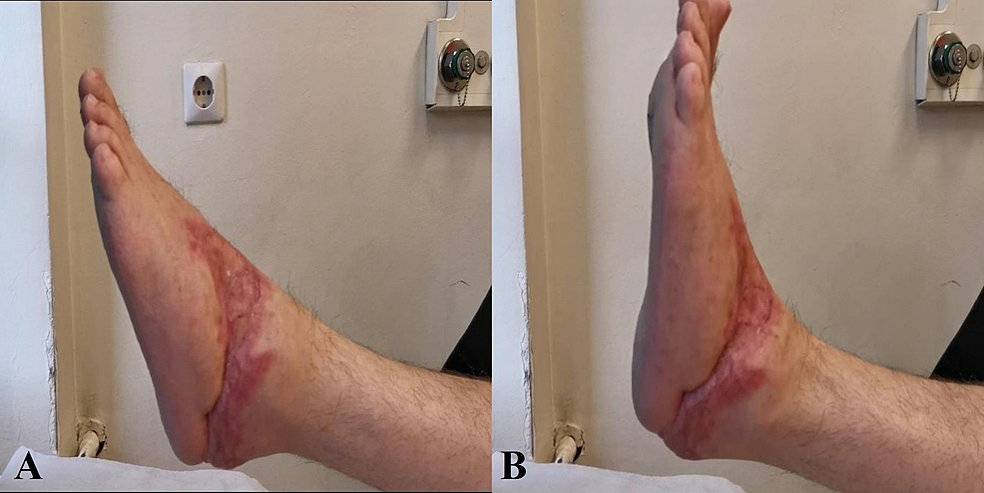
Range of motion eight months post trauma (full plantarflexion [A]/dorsiflexion[B])

**Figure 8:**
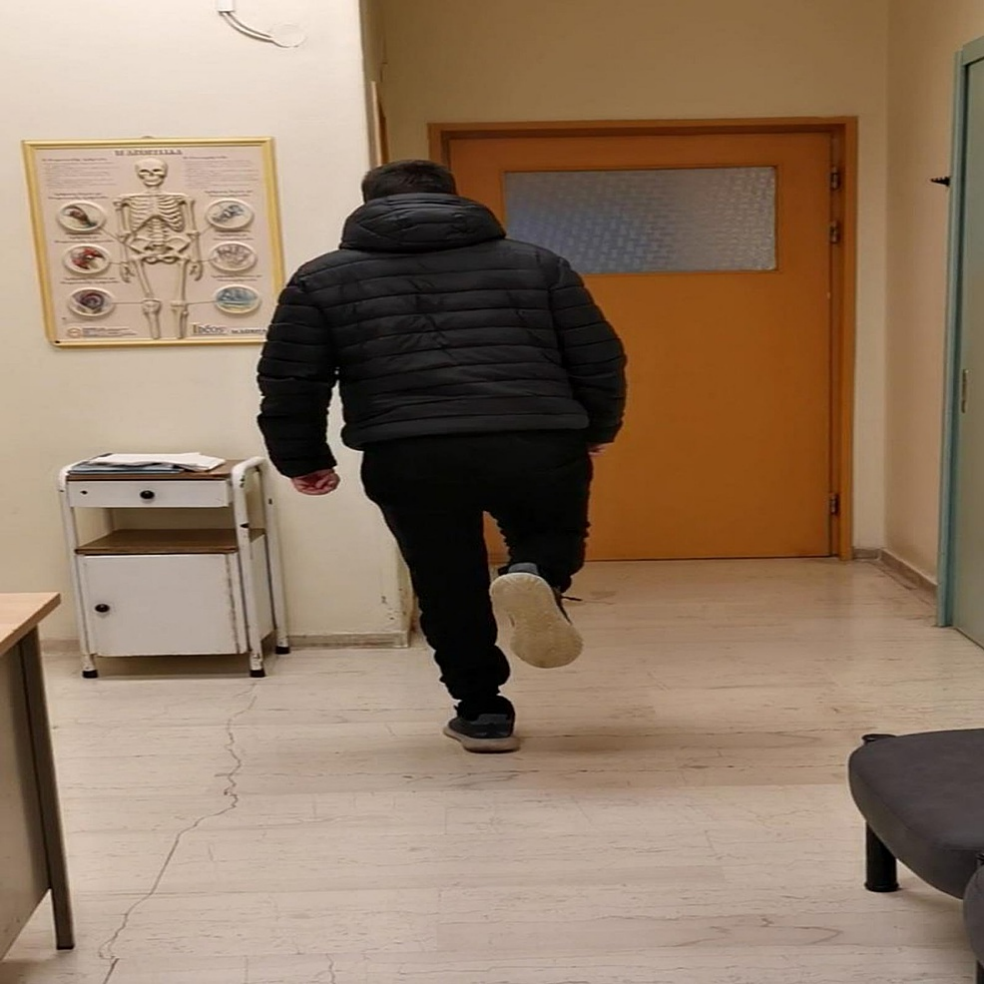
One leg stance test eight months post-op.

At 10 weeks post-injury, instructions for partial weight-bearing were provided and at three months full weight-bearing was recommended. At that time, clinical examination and radiographs demonstrated a full recovery of the injury. At six months, the patient, following a standardized accelerated rehabilitation protocol, displayed a nearly normal range of motion, the deficit in sensation over the dorsal side of forefoot but intact sensation over his sole and ambulated independently, wearing regular shoes without pain (Figures 6-8).

 

 

## Discussion

Despite the progress that has been made in soft-tissue reconstruction over the last decades, degloving injury remains a challenging condition to treat [2,4]. Regarding the management of such complex traumatic cases, every therapeutic protocol that has been demonstrated aims to save the limb with minimal postoperative morbidity [2-4]. Typical treating options for maintaining the congruity and viability of soft tissue structures involve reattachment of the avulsed skin, negative-pressure-wound-therapy, split- or full-thickness skin graft and use of regional or free flaps [1-5]. However, there is no certain algorithm for each patient and thus, each case should be considered unique. The low survival rate of skin grafts and donor site morbidity along with insufficient availability of flaps especially in cases of both osseous and large soft tissue defects, limit the possibility for a good clinical outcome [4-7].

In our case, the patient suffered from a complex degloving injury at his left foot which mainly concerned his heel pad. Apart from the separation of soft tissues from the deep fascia and bones, the patient displayed neurovascular damage, tendon ruptures and hindfoot and midfoot fractures.

The literature regarding such heel pad degloving injuries is rather limited. In general, the majority of studies reported poor prognosis leading mostly to amputations particularly when there is a complete subcutaneous detachment [8-10]. This could be attributed to the anatomy of the heel pad, which is composed of a thick layer of adipose tissue confined by fibrous septa that protects the foot from stress generated during ambulation [10]. The plantar surface of the foot is hard to be covered by skin grafts or flaps as they cannot offer the required resilience for ambulation [1,3,10]. Therefore in cases of partial heel pad degloving injury with intact vascularity, attempts should be made to maintain the avulsed skin [8].

Particularly, regarding the vascular supply to the heel pad, the integrity of the lateral calcaneal branch of the peroneal artery and medial calcaneal branch of the posterior tibial artery is considered crucial for healing after heal pad avulsion injuries [4]. On the contrary, there are studies that report successful heel pad replantations using skin grafts or flaps [4-7]. The vascular damage in our patient concerned only the dorsalis pedis artery whereas the calcaneal branches to the heel pad were intact.

Furthermore, osseous defects or dislocations requiring fixation are negative prognostic factors in degloving injuries. Only a few cases report degloving injuries where internal or external fixation was performed for fracture stabilization [2,3,5]. On the contrary in our study, no fixation system was used to stabilize the fractures in the calcaneus, cuboid and cuneiform bones.

In addition, comorbidities such as peripheral vascular disease, diabetes, smoking, or malnutrition might have an adverse impact on the final clinical outcome [11]. Our patient was young without any medical history and aside from smoking, there was no other compounding element that could affect the recovery.

The role of negative-pressure-wound therapy in managing severe soft tissue injuries with successful functional results, preventing limb amputation, has been well established [12-14]. VAC accelerates wound healing not only by increasing blood flow but also by suctioning exudates and eradicating bacteria [15]. Thus, a reduction in the size of skin defects and formation of granulation tissue is achieved, which is essential for secondary soft tissue reconstruction procedure [16]. In our patient, the rejuvenating effect of negative-pressure-wound therapy was critical for the successful healing process.

## Conclusions

Although each degloving heel pad injury is unique and it is hard to propose one single protocol to manage every case, the combinatorial role of negative-pressure-wound therapy and consequent use of split-thickness graft might be determinant factors in the strategic management to achieve a functionally and cosmetically acceptable long-term outcome.
